# Acceptability and Preferences of Two Different Community Models of ART Delivery in a High Prevalence Urban Setting in Zambia: Cluster-Randomized Trial, Nested in the HPTN 071 (PopART) Study

**DOI:** 10.1007/s10461-021-03385-8

**Published:** 2021-07-24

**Authors:** Mohammed Limbada, Chiti Bwalya, David Macleod, Osborn Shibwela, Sian Floyd, Diana Nzara, Vasty Situmbeko, Richard Hayes, Sarah Fidler, Helen Ayles, the HPTN 071 (PopART) Study Team

**Affiliations:** 1 Zambart House, School of Medicine, University of Zambia, Ridgeway Campus, Off Nationalist Road, P.O. Box 50697, Lusaka, Zambia; 2 Imperial College, United Kingdom and Imperial College NIHR BRC, London, UK; 3 Department of Infectious Disease Epidemiology, London School of Hygiene and Tropical Medicine, London, UK; 4 Department of Clinical Research, London School of Hygiene and Tropical Medicine, London, UK

**Keywords:** HIV, Anti-retroviral therapy, Home-based ART delivery, Adherence clubs, Stated preference

## Abstract

Community delivery of Antiretroviral therapy (ART) is a novel innovation to increase sustainable ART coverage for People living with HIV (PLHIV) in resource limited settings. Within a nested cluster-randomised sub-study in two urban communities that participated in the HPTN 071 (PopART) trial in Zambia we investigated individual acceptability and preferences for ART delivery models. Stable PLHIV were enrolled in a cluster-randomized trial of three different models of ART: Facility-based delivery (SoC), Home-based delivery (HBD) and Adherence clubs (AC). Consenting individuals were asked to express their stated preference for ART delivery options. Those assigned to the community models of ART delivery arms could choose (“revealed preference”) between the assigned arm and facility-based delivery. In total 2489 (99.6%) eligible individuals consented to the study and 95.6% chose community models of ART delivery rather than facility-based delivery when offered a choice. When asked to state their preference of model of ART delivery, 67.6% did not state a preference of one model over another, 22.8% stated a preference for HBD, 5.0% and 4.6% stated a preference for AC and SoC, respectively. Offering PLHIV choices of community models of ART delivery is feasible and acceptable with majority expressing HBD as their stated preferred option.

## Introduction

By the end of 2016, 36.7 million [30.8–42.9 million] people were living with HIV globally with the vast majority living in low-and middle-income countries [1]. East and Southern Africa are the most affected regions with approximately 19.4 million People living with HIV (PLHIV) accounting for more than half the world’s HIV-positive population [1]. As the world commits to reaching the UNAIDS 90-90-90 targets for HIV diagnosis, treatment, and viral suppression by 2020 to end the AIDS epidemic by 2030, there has been substantial progress in scaling up Antiretroviral treatment (ART) programs globally. By 2018, more than 2/3 (79%) of all PLHIV knew their status with 23.3 million (62%) PLHIV accessing ART [2].

Zambia has an estimated HIV prevalence of 11.3% in adults with an estimated 1.2 million PLHIV [3], most in urban areas. The government, in close partnership with its PEPFAR implementing partners has made tremendous progress over the last decade towards epidemic control with 63% of Zambians in need of ART receiving it by the end of 2016 [3–6]. Following the adoption of the WHO [8] treatment guidelines there has been an expansion in numbers of people on ART to about 85% of PLWH who know their status [7–10]. In spite of these successes, the ART treatment program in Zambia still faces many challenges. From the demand point of view, the change in guidelines increased the demand for ART services resulting in overcrowding, long waiting times and overburdening of the already fragile health facility system thus increasing the workload and burnout for the few existing health care workers [11, 12]. These challenges compromise service delivery and may lead to ART interruption, poor adherence, ongoing transmission and the development of viral drug resistance and increased mortality [9, 10, 13]. Adherence to treatment and virological suppression are critical for survival and prevention of onward viral transmission and without a change in the current delivery model of ART in Zambia, lifelong ART for all PLHIV is unsustainable. Decentralization of ART services into the community through community-based ART delivery models is one of the WHO recommended strategies to maintain the HIV continuum of care in resource limited settings where the existing formal health systems are unable to cope with increasing numbers of PLHIV on treatment [13].

Community-based models decentralize HIV services leading to improved service delivery by reducing congestion at health facility, maintaining capacity of clinic staff and enhancing access to ART and adherence for PLHIV allowing them to have more power to coordinate their lives between treatment and livelihood options. These models have proven to be effective in a number of settings, empowering patients on ART and communities to take responsibility for their own treatment [14, 15]. In many settings across sub-Saharan Africa (SSA), these models have shown promising outcomes in relation to retention and adherence to treatment and strengthening community engagement by linking community based programs with the existing health care facilities [13, 16–18]. These models are designed for “stable” patients, i.e., those on suppressive ART, to receive HIV services in the community thus reducing frequent clinic and pharmacy visits, transport costs and long waiting times and allowing the health care facility to focus more on patients with advanced disease. The models have been divided into four categories: health care worker-managed group models; client-managed group models; facility-based individual models; and out-of-facility based individual models [19].

The recently ended HPTN 071 (PopART) trial was a community randomized trial conducted in 21 urban communities in Zambia and South Africa investigating whether a community-wide combination HIV prevention package including home-based HIV testing, linkage to care and immediate ART for those who test positive will help reduce HIV incidence [20, 21]. Early data from the PopART intervention in Zambia showed that there were delays in initiation of treatment [21]. Community based approaches to ART delivery have the potential to improve linkage and retention in care and hence help bridge this gap. The design of the PopART intervention, where universal door to door HIV services were provided by trained Community HIV providers (CHiPs), provided us with a unique opportunity to pilot different models of ART delivery with support from the Zambian Ministry of Health. Despite several pilot programs having achieved remarkable success across many settings in sub Saharan Africa [22–24], much about the comparisons between community models of ART delivery and conventional facility-based delivery has been in relation to retention in care and on treatment and the frequency of clinical visits. Very few have compared different models of ART delivery with each other, in particular through soliciting patient preferences for different models of ART delivery [25]. In addition to providing evidence on long term outcomes, cost effectiveness, uptake and acceptability of different models of ART delivery, determining patient preferences towards these models will allow national HIV programs to design and implement models of ART delivery that work best and most appealing to patients in various settings.

To this effect, a three-arm cluster-randomized non-inferiority trial was nested in two of the HPTN 071 (PopART) trial intervention communities with the aim of evaluating clinical outcomes, feasibility and effectiveness of two community models of ART delivery (Adherence clubs and Home-based delivery) for stable HIV+ patients in an urban high HIV prevalence setting in Lusaka, Zambia. In this paper we describe choices (“revealed preferences”) and stated preferences of PLHIV for ART delivery models outside the health facility.

## Methods

### Study Design and Participants

The study was nested in two communities that had been part of the HPTN 071 (PopART) trial. Details of the main HPTN 071 (PopART) trial have been described elsewhere [26]. We conducted a three-arm cluster-randomized non-inferiority trial to compare virological suppression at 12 months in stable HIV+ patients receiving ART between individuals allocated to receive either ART via community models of ART delivery and those receiving in the facility-based (standard of care). The two Lusaka communities chosen for this sub-study resembled other PopART communities in Zambia and reflected the situation with respect to clinic burden HIV prevalence and population migration for many sub-Saharan African countries urban settings in resource limited settings.

The two communities selected for this cluster randomized trial were matched by community size and HIV prevalence (18% and 21% amongst adults aged 18–44). Each community was divided into geographical zones of approximately 500 households (approximately 1400 individuals) and each zone was managed by a pair of trained Community health workers (CHWs). There were 104 zones across the two communities.

All adult HIV+ patients (≥ 18 years) defined as “stable” in accordance with WHO definitions [19] residing in the two urban communities, enrolled for ART in the two primary health care facilities serving the communities, were eligible for inclusion in this nested study. WHO classification for “stable “patients, was (1) Taking first line ART for at least 6 months, (2) Virally suppressed according to national guidelines, and (3) Had no other health conditions requiring the attention of a clinician. An additional eligibility criterion for our study included patients living within the HPTN 071 catchment area and being willing to provide written informed consent.

### Randomization

The 104 zones across the two communities were randomly assigned (35:35:34) to one of the three study arms for ART delivery. The three study arms were: Arm 1. Facility-based ART delivery (Standard of care, (SoC) continued collection of ART only at the health care facility, Arm 2. Home-based ART delivery (HBD) where ART was delivered to the participant’s home every 3 months by a community health worker (HCW) and Arm 3. Being part of an Adherence club (AC), meeting every 3 months outside of the health care facility and facilitated by a community health worker. In the HBD and AC arms participants were given the choice to continue with ART delivery through the health care facility or to accept the community model of ART delivery route they had been allocated. To achieve balance across the clusters, we stratified randomization by community and further restricted the randomization, first within each community and second across both communities on average values of key outcomes including population size, HIV prevalence which was available at the entire community level only, and proportion of HIV+ patients who attend the local health care facility and distance to the health care facility to ensure overall balance across the study arms.

A public randomization ceremony was held with the community health workers, their supervisors, the primary health care staff, members of the PopART intervention study teams and community advisory boards to allocate the zones to one of the three study arms.

### Study Outcomes: Participant’s ART Delivery Choices (Revealed Preference) and Stated Preferences

This study explores the participants ART delivery preferences, these have been divided into stated and revealed preferences; stated preferences are those which people say they want and revealed preferences are what they actually choose. The community models of ART intervention were implemented between 3rd May 2017 and 30th April 2019. All eligible patients attending the health care facility were invited to join the study and were asked to consent. Having consented to the study, participants were assigned to a study arm. Those who were assigned to the two community models of ART delivery arms were first asked to choose between the assigned interventions and or continue with facility-based care (SoC), their revealed preference. Participants who were assigned to the facility-based (SoC) arm did not have an option to choose. Subsequently all participant’s (including those in the SoC) were asked “*did you have a preferred model of ART delivery out of the three options? If yes, which model of delivery?”*. The response to this question we define as the participant’s stated preference and can be one of the four categories: (1) Prefers Facility-based (SoC); (2) Prefers Home Based ART delivery; (3) Prefers Adherence clubs and (4) No preference expressed.

In this paper we describe the revealed preferences made by those in the two community models of ART delivery arms (as to whether they chose facility-based (SoC) or the allocated community model of ART delivery) and the stated preference of all participants about the different models of ART delivery. The stated and revealed preferences were recorded on the enrolment form by the study staff. Participant characteristics with regard to age, sex and years on ART were also collected as part of the general survey during enrolment. All data were entered into an electronic data collection system.

### Statistical Analysis

STATA version 13 was used to clean and analyse the data. Descriptive data on the study participants reported preferences were stratified by study arm and presented as medians and interquartile ranges for the continuous variables and proportions for the categorical variables. Of patients who consented to participate in the study, we determined the proportion who chose the model of delivery assigned to them and the proportion of participants who stated a preference for each model of ART delivery (or no preference). We further conducted analysis by sex, age group, years on ART and trial arm to explore whether there were any associations between these variables and stated preference using Pearson’s Chi square test.

An exploratory qualitative study using observations, interviews and Focus group discussions (FGDs) was used to collect qualitative data. Observations of Home based model delivery (HBM) (n = 12) and Adherence club meetings (n = 6), audio-recorded in-depth interviews with a purposively selected sample of PLWH accessing ART through the two models (n = 27) and two FGDs with community health workers administering the models (n = 16) were conducted eight months after the start of the intervention between October and December 2017 and at the end of the study between May and August 2019. Observations provided insights into how community health workers conducted the delivery of ART as well as the micro-social environment surrounding clients. Interviews and FGDs inquired about preferences and experiences of PLWH with accessing ART and acceptability. All discussions ended with participants plotting their overall opinion of the models on a simple visual scale with different facial expressions (emoji’s) corresponding to degrees of satisfaction and acceptability.

All audio recordings from FGDs and IDIs were transcribed verbatim and translated to English by the second author. Notes taken during and after the observation were expanded and typed in Word and then later on saved with a unique code representing each participant. All data transcripts including typed notes were imported into Atlas.ti 7 and using the Thematic coding analysis (TCA) approach, all parts of the data transcripts were subjected to iterative coding process by the first author [27, 28]. Analytical categories of related codes and sub codes were then stratified by study site and participant profile. Using Atlas ti 7, code outputs [codes linked to quotations from transcripts and summed up in a theme] were created representing recurrent themes related to factors influencing choice of a model and acceptability and served as a basis for further analysis and interpretation.

### Ethics

The study was granted ethical clearance from the University of Zambia Biomedical Research Ethics (UNZABREC) and the London School of Hygiene and Tropical Medicine ethics committee. Prior to approval, this protocol had also been through regulatory review and approved by Division of AIDS (DAIDS) who granted us permission to carry out this study as an ancillary study to HPTN 071 (PopART) and registered at ClinicalTrials.gov. Further approvals were granted by Zambian National Health Research Authority and Ministry of Health. Written informed consent was obtained from all participants.

## Results

**Fig. 1:**
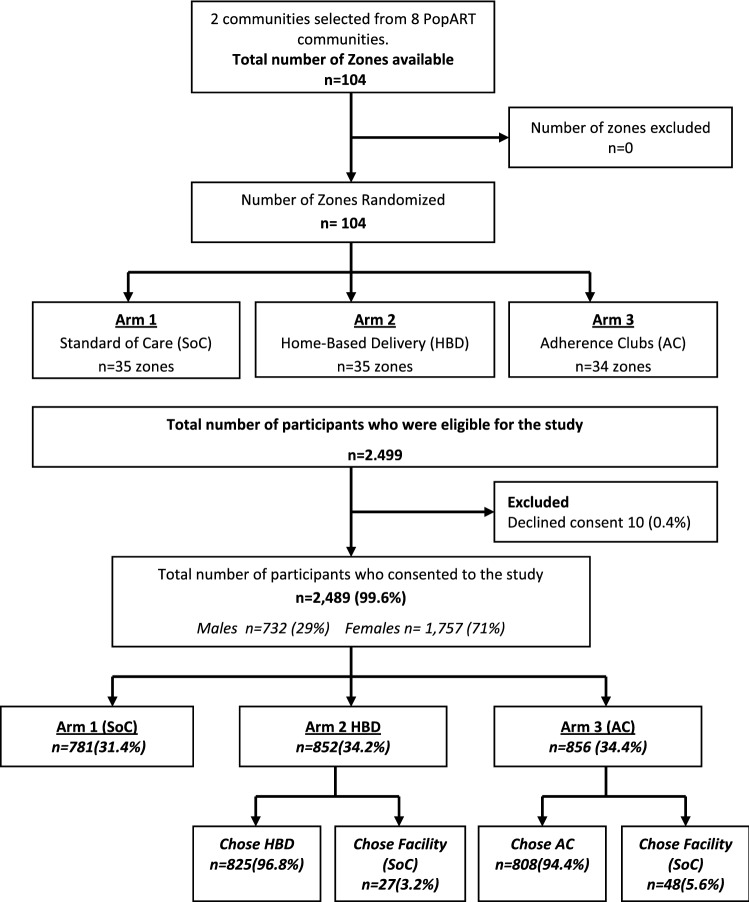
Uptake and choices across the three study arms. Data are n (%)

A total of 2499 eligible participants were identified across the two communities between May and December 2017 who were eligible for inclusion in the trial and of these, 2489 (99.6%) consented to participate, 10 (0.4%) declined consent (Fig. 1). The three study arms were well balanced according to baseline characteristics, However there were fewer participants in the SoC arm. Of the participants who were eligible, the majority were female across all arms (N = 1757, 71%), reflecting the stable patient clinic population on ART. The median age of participants was 40 years (IQR: 33–47) in the SoC and AC arms and 39 years (IQR: 33–46) in the HBD arm. The median years being on ART was 4 years (IQR: 2–7) across all three arms.

### Choices of Models of ART Delivery in the Two Community Models of ART Delivery Arms

There were 781 (31.4%) stable participants were assigned to the SoC arm, 852 (34.2%) assigned to HBD arm and 856 (34.4%) to AC arm. Of the participants who were assigned HBD, 27 (3.2%) chose to continue receiving care at the clinic and 48 (5.6%) who were assigned AC chose to continue receiving care from the clinic. Among the participants randomized to the community models of ART delivery arms [HBD and AC], overall, 95.6% chose the community models of ART delivery that they were randomized to receive [96.8% in the HBD arm and 94.4% in the AC arm] (Fig. 1).

### Preferences for Models of ART Delivery

Out of the 2489 participants who were asked for their stated preference of ART delivery model, 1682 (67.6%) did not state a preference of model of delivery over any of the others, 568 (22.8%) stated they would prefer home-based delivery, 125 (5.0%) stated they would prefer adherence clubs and 114 (4.6%) stated a preference for facility-based (SoC) (Table 1). Participants in the facility-based (SoC) arm were most likely to state a preference of one mode of ART delivery over another and of those that did state a preference the majority preferred HBD. Overall in the SoC arm, 39% stated no preference, 48% HBD, 12% AC and only 1% preferring SoC. Few individuals in the HBD arm stated a preference of one model of ART delivery over another, with 88% reporting no preference, and among those who did state a preference they preferred SoC over the two community models of ART delivery options. In the AC arm, 73% did not state a preference but amongst those that did there was a clear preference for HBD, with 19% stating this preference (Table 1). We found no evidence of association between stated preference for ART delivery model and any of age, sex or time on ART, but did find strong evidence (p < 0.001, χ^2^(6) = 670.4) of an association with trial arm (Table 1). Among the 27 participants in the HBD study arm who chose to receive SoC, all 27 stated a preference for SoC when asked. However, in the same arm, an additional 19 participants chose HBD despite their stated preference being for facility-based (SoC). Similarly in the AC arm out of the 48 participants who chose SoC, 46 participants stated a preference for SoC, one a preference for HBD and one had no preference, an additional 11 participants who stated a preference for SoC chose to join AC anyway (Table 2).

**Table 1:** Participants stated preferences for model of ART delivery and associations between preferences and participant baseline characteristics

	Overall (n = 2489)	No preference	Preferred SoC	Preferred HBD	Preferred AC	P-value*
1. Trial arm		N = 1682 (67.6%)	N = 114 (4.6%)	N = 568 (22.8%)	N = 125 (5.0%)	***P* < *0.001*** χ^2^(6) = 670.4
Standard of care	781	303 (38.8%)	9 (1.2%)	377 (48.3%)	92 (11.8%)	
Home based delivery	852	751 (88.1%)	46 (5.4%)	25 (2.9%)	30 (3.5%)	
Adherence clubs	856	628 (73.4%)	59 (6.9%)	166 (19.4%)	3 (0.4%)	
2. Sex						
Male	732 (29.4%)	489 (66.8%)	43 (5.9%)	157 (21.4%)	43 (5.9%)	***0.101*** χ^2^(3) = 6.2
Female	1757 (70.6%)	1193 (67.9%)	71 (4.0%)	411 (23.4%)	82 (4.7%)	
3. Age group						
18–24	111	82 (73.9%)	4 (3.6%)	20 (18.0%)	5 (4.5%)	***0.713*** χ^2^(12) = 8.9
25–34	610	422 (69.2%)	25 (4.1%)	135 (22.1%)	28 (4.6%)	
35–44	992	668 (67.3%)	53 (5.3%)	224 (22.6%)	47 (4.7%)	
45–54	554	360 (65.0%)	26 (4.7%)	134 (24.2%)	34 (6.1%)	
55+	222	150 (67.6%)	6 (2.7%)	55 (24.8%)	11 (4.9%)	
4. Years on ART						
< 1 year	77	47 (61.0%)	8 (10.4%)	16 (20.8%)	6 (7.8%)	***0.189*** χ^2^(9) = 12.5
1–2 years	671	462 (68.8%)	32 (4.8%)	144 (21.5%)	33 (4.9%)	
3–5 years	829	563 (67.9%)	40 (4.8%)	192 (23.2%)	34 (4.1%)	
> 5 years	912	610 (66.9%)	34 (3.7%)	216 (23.7%)	52 (5.7%)	

Data are n (%)

*Pearson’s Chi square test

**Table 2:** Preferences amongst those who chose the community models of ART delivery versus those who did not

	Home-based delivery arm		Adherence club arm	
Stated preferences	Chose SoC	Chose HBD	Chose SoC	Chose AC
No preference	0	751	1	627
Preferred standard of care	27	19	46	13
Preferred home-based delivery	0	25	1	165
Preferred adherence clubs	0	30	0	3

Qualitative findings from in depth interviews with 27 study participants, revealed a number of factors that may have influenced participants’ preferences for HBD compared with AC and SoC. Congestion at the clinic was reported to be a prominent factor that may have influenced the preference for out of facility models, especially HBD. For all PLHIV interviewed, overcrowding of the ART clinic was the major reason why they preferred HBD. As one male participant reflected: ‘The issue of having too many people at the clinic was a real problem that made the waiting worse.’ ‘Congestion is a real and big problem at the clinic’, added another female participant*.* CHWs also mentioned overcrowding at the clinic was the main reason that made people prefer HBD. In addition, there were a number of other challenges at the clinic that may have influenced the choice of HBD followed by AC. The waiting area at the health facility was said to be very small, with only a few benches that could not accommodate all clients meaning most had to stand in the sun for a very long time while they waited to be attended to. Moreover, the location of these waiting areas was a problem for some patients because they feared being seen by others while they were waiting at the separate ART clinic building.

“ … They say it is not fair that they are separated from the rest of the clinic… I have two clients, for them they even said they have even stopped coming to the clinic. ‘When going to get my drugs, the location of clinic makes everyone to see you immediately you enter, and they will know that you have gone to get ARVs”. (FGD, CHWs, community 1).

The culture of clinic staff was also cited as reason as to why people preferred the HBD and the AC models. PLHIV complained of harsh treatment by health care workers. Being shouted at and the use of stigmatizing language was a commonly reported experience by PLHIVHs as reported by CHWs:

“Others felt as if they were not treated with dignity and respect, which caused them to get frustrated. If someone calls you name and you do not respond, you will be shouted at, ‘eh, ‘we are calling you and you are not answering, did you not hear your name being called’. So, those clients started feeling frustrated. So, when we came in [referring to CHiPs and the Model], they started saying that this was easy” (CHWs, FGD, community 1).

Long waiting time was another contributing factor as PLHIV reported coming to the clinic as early as 05.30am to have access to treatment early and then go for work on time.

“It was hard because there used to be huge groups of people at the clinic, when you go at 6 AM, you come back around 14–15PM.” (IDI, woman, club member, community 1).

For patients to avoid long queues and receive drugs more quickly, an informal trade between patients and clinic staff, especially lay counsellors, was established. Participants mentioned that for them to skip the queue and be attended to faster, they could pay staff from 10 kwachas ($1) to 50 kwachas ($10), with the amount to be paid being dependent on the economic status of the client. Once this was done, the staff would then find means for a patient to be attended to in the quickest manner possible. This informal trade was perceived as adding to the waiting times for those patients that did not have money to pay the staff. Those that paid could come late and be seen before those that came early. This informal and hidden arrangement was considered to have become part of the organizational routines of the clinic.

“If you wanted to be attended to quickly, just pay a K50. In addition, it has actually become a routine, because for those that pay, they will spend at least an hour and then leave. But for those who don’t, they are likely to spend the whole day there” (CHWs FGD, community 2).

The HBD model and, to a large extent, the AC had several advantages over the clinic. Participants described their experiences with the HBD and AC model as the opposite of that of the clinic. They reported the overriding advantage of HBD and AC being the convenience and control that participants were able to retain over their time with respect to their livelihood activities as most of them worked in the informal sector which required them to leave home early in the morning and come back late in the evenings. The community ART models made it possible for them not to have to choose between going to the clinic and going to work. Drugs were delivered at prearranged times through an appointment system, enabling PLHIV to plan their work or business activities around this. The practice of CHWs making and re-confirming appointments with clients allowed them the mobility they required to continue livelihood activities. HBD was considered better than AC because drugs were delivered in the homes and participants did not have to move from their homes to a communal venue unlike the clubs.

Another factor cited by participants was the fact that the models were new HIV initiatives delivered by trusted counsellors that people already knew. The CHWs were well known within the communities and had built relationships of trust with household members during the course of the main trial. When the HBD model was implemented, participants were free to choose it because they knew the people that were supposed to be delivering the intervention as one CHWs reflected:

“They are welcoming, because we’ve been with them and we have created that rapport from the beginning. So, they know us” (CHWs FGD, community 2).

In all the observed home visits by CHWs, clients seemed very happy with the visit, greeting the CHWs with smiles. During the interviews, participants were asked about their view on the models and asked to plot their overall opinion of the HBD and AC on a simple visual scale with different facial expressions (emoji’s) corresponding to the degrees of satisfaction from not at all satisfied to extremely satisfied. The majority of PLHIV gave the model a score of 5/5, indicating they were extremely satisfied with the way the model had fit into their lives. This satisfaction is reflected in the following quote from one of the participants.

“Well, I am very happy with this programme and everything that happens in it. It has reduced the problems I used to face when I used to go to the clinic, making us stand in queues, leaving the clinic late; it has reduced all of that.” (IDI, Woman, Community 2).

When comparing the number of study participants that said they were either very satisfied or extremely satisfied with the two models, the HBD had more PLHIV expressing high levels of satisfaction than those that were from AC. A few participants rated the model four out of five indicating they still faced problems with follow-up procedures at the facility whilst others were neutral about the clubs because they felt the clubs should rather meet every 6 months than 3 months to give them enough time for their livelihood activities.

Both HBD and AC were seen as models that reduced the stigma previously experienced at the clinic. Accessing ART from dedicated areas in the local clinic was interwoven with fears about *‘being seen accessing ART’* and being recognised as a person with HIV. Establishing the HBD on the back of a door-to-door HIV testing programme and using the same people (CHWs) was mentioned by the majority of PLWH as one major factor that minimized stigma during the home visits. The existence of the prior programme helped veil the delivery of ARVs as everyone identified the CHWs with the HIV testing programme and not with the ART delivery programme.

‘My neighbours do not know the reason why they visit me but what they do know is that they move door to door in each and every household checking on people.” (IDI, woman, community 2).

## Discussion

This manuscript describes the choices and stated preferences of models of ART delivery amongst a group of stable people living with HIV who consented to enrol into a CRT comparing acceptability and safety of two different models of delivering ART outside of current facility based care with the standard of care. In our study population, over 95% chose the community models of ART delivery rather than facility-based (SoC) when offered a choice (as their “revealed preference”). When asked to express their preference for a mode of ART delivery, over 65% did not state a preference but for those who stated a preference, there was an overwhelming acceptance and enthusiasm for community models of ART delivery options.

Our findings confirm that decentralizing ART care outside the current facility-based care into the communities using community health workers to provide adherence support and pre-packed medications is feasible and acceptable. This is consistent with findings from previous studies which have shown that community-based ART programs can achieve remarkable results in expanding access to treatment and retention in resource limited settings [29] as they overcome many of the challenges patients face such as long waiting times to access medications, frequent clinic trips and transportation costs [30–33]. An analysis of programmatic data by Broad Reach International [34] from 217 facilities in five districts in South Africa between 2016 and 2017, showed rapid uptake of differentiated models of care (facility and out-of-facility based) with approximately 75% of eligible patients accepting and a 10% increase in patients moving to community based models. However, it is unknown whether patients in this analysis were offered a choice between the models and standard of care. Similarly, the HPTN 071 (PopART) trial showed that using Community HIV providers (CHiPs) to provide a door-to-door combination HIV package is well accepted, feasible and effective in coverage of HIV testing and knowledge of status in both adults and adolescents [35, 36]. To-date there is limited data documenting patient choices and preferences towards models of ART delivery in resource-limited settings as well as factors associated with the choices patients make towards non-facility based care although location-related preferences appeared prominent [37], with most patients citing long waiting times, overcrowding and distance as the real reason for their choice. Findings from a recent study in Zambia using discrete choice experiments to ass stable HIV patient’s preferences towards differentiated care models demonstrated substantial heterogeneity with the strongest overall preference for reduced clinic visits [25].

Exploring choices and preferences that patients make towards health care in resource limited settings is difficult and there is very limited data on health decision making by patients in these settings. A review of literature has revealed that patient involvement in health care decision making is empowering and has been associated with improved treatment outcomes [38]. Recognition of a patient’s knowledge, health care worker- patient relationship, allocation of sufficient time for participation and also factors associated with patient’s knowledge, beliefs, physical and cognitive abilities and values can influence patient participation in health care decisions [38]. In resource limited settings, health care workers with constrained resources are unable to offer a choice and instead dictate to the patient who are therefore unused to being asked to express their own choices and usually do not, for fear of being neglected in care. Patients may struggle to choose between health care options as they lack confidence, may not be sure of the options they would prefer or have conflicting priorities [39]. In resource-constrained settings, patient’s choices tend to be influenced by structural aspects of the health care service such as availability and accessibility of health care providers, quality of staff, costs of treatment and by processes such as availability of information, continuity of treatment, waiting time and transport costs [40]. This could explain the reason why patients would “choose” the interventions offered to them.

In our study population, over 95% of the participants in the community models of ART delivery arms who were offered a choice between community and facility-based care options chose the former. It may be understandable that barriers such as location, distance to clinic, overcrowding and long waiting times are important factors and whilst these barriers are well known determinants of uptake, acceptability and outcomes [41], reasons for choosing community models of ART delivery may vary. When determining patient preferences, 1/3 of our study population stated a preference towards a model of ART delivery and of these, majority stated a preference for community models of ART delivery (HBD and AC) compared to SoC. A large proportion, 2/3 of the study population did not state a preference towards any of the 3 ART delivery options and whether that reflects a true lack of preference needs to be explored further. Some of the possible explanations as to why our study participants were unwilling to state a preference could be that: (1) They do not perceive themselves as having much autonomy of choice when it comes to health care services; (2) They are not empowered about choice especially in resource-limited and (3) The design of our study where participants were assigned to the study arm before they were asked on their preferences and therefore less likely to state a preference when satisfied with what they had received, for example, only 11.9% of participants in the HBD arm actually stated a preference compared with 61.2% in the Soc arm. In the study arms where participants did not get an option off being in HBD, a higher stated preference towards HBD was observed over the other two options. In both HBD and AC arms, participants were less likely to state preferences towards the modes of ART delivery.

Although several studies have suggested increasing experience of stigma by household members who were receiving follow-up visits by community health workers to link to care when tested positive [42], it appears that in our study, the high acceptability of community models of care by participants did not perceive community health workers providing HIV support and drug delivery in their homes or community venues as stigmatizing. This could largely be due to the fact that during the HPTN 071 (PopART) trial, repeated home visits by community health workers delivering the door-to-door combination prevention package over the course of 3 years solidified the relationship between CHWs and the communities and could also have changed the communities perception towards these cadres [35, 43]. However, we cannot say how these findings may be generalizable to other settings that do not have community workers delivering HIV interventions.

The study had a number of limitations. First this study was done in an urban setting where patients have never been exposed to community models of ART delivery or other forms of differentiated care offered by the government as part of their health care services, and therefore may not have been able to or could have struggled to determine their preferences towards community models of ART delivery unknown to them. Secondly, the design of our study where participants were asked for their preference only after they were assigned to the study arms could have led to bias. It is possible that had we asked for their preferences prior to them knowing which mode of ART delivery they were being allocated to, we may have found a different outcome.

Our review sheds light for future opportunities to conduct preference studies in resource-limited settings. As HIV programs scale up community models of ART delivery in the context of universal treatment, there is need to further identify patient and provider preferences for community models of care that will improve clinical outcomes.

## Conclusion

Offering PLHIV a choice of different models of ART care in high-burden resource limited settings is possible and when offered a choice between facility and community models of ART delivery, the majority of those who expressed a preference stated a preference for home-based ART delivery, the revealed preference when the option was implemented was over 95%. As national programs scale up models of ART delivery in resource- limited settings, acceptability, choices and preferences will be important in order to determine which models to prioritize as they could be significant factors in clinical outcomes and integrity of the models of delivery.

## Data Availability

Research data that underpins analysis outlined in this paper cannot be made available. The dataset contains measurements that may be used to re-identify study participants, due to the number and type of variables captured. Participant and ethical consent for wider sharing was also not obtained, due to the research being performed at a time prior to data sharing becoming the norm. However, the study team invite interested parties to contact them to discuss the research and data collected in further detail.
